# Comparison of remimazolam tosilate and propofol during induction and maintenance of general anesthesia in patients undergoing laparoscopic cholecystectomy: a prospective, single center, randomized controlled trial

**DOI:** 10.1186/s12871-024-02614-6

**Published:** 2024-07-06

**Authors:** Zhuxin Luo, Hai Cao, Li Luo, Long Chen, Dian Feng, Guihua Huang

**Affiliations:** The Third Affiliated Hospital of Zunyi Medical University, (The First People’s Hospital of Zunyi), Guizhou, China

**Keywords:** Remimazolam tosilate, Propofol, Total intravenous anesthesia

## Abstract

**Background:**

Remimazolam tosilate (RT) is a new, ultrashort-acting benzodiazepine. Here, we investigated the efficacy and safety of RT for general anesthesia in patients undergoing Laparoscopic Cholecystectomy (LC).

**Methods:**

In this study, 122 patients undergoing laparoscopic cholecystectomy were randomly allocated to receive either remimazolam tosilate (Group RT) or propofol group (Group P). RT was administered as a slow bolus of 0.3 mg kg^− 1^ for induction, followed by 1.0–2.0 mg kg^− 1^ h^− 1^ for maintenance of general anesthesia. Propofol was started at 2 mg kg^− 1^ and followed by 4–10 mg kg^− 1^ h^− 1^ until the end of surgery. The primary outcome was the time to bispectral index (BIS) ≤ 60. The secondary outcome included the time to loss of consciousness (LoC), and the time to extubation. Adverse events were also assessed.

**Results:**

A total of 112 patients were recruited for study participation. Among them, the time to BIS ≤ 60 in Group RT was longer than that in Group P (Group RT: 89.3 ± 10.7 s; Group P: 85.9 ± 9.7 s, *P* > 0.05). While the time to LoC comparing remimazolam and propofol showed no statistical significance (Group RT: 74.4 ± 10.3 s; Group P: 74.7 ± 9.3 s, *P* > 0.05). The time to extubation in Group RT was significantly longer than that in Group P (Group RT: 16.0 ± 2.6 min; Group P: 8.8 ± 4.3 min, *P* < 0.001). Remimazolam tosilate had more stable hemodynamics and a lower incidence of hypotension during general anesthesia.

**Conclusions:**

Remimazolam tosilate can be safely and effectively used for general anesthesia in patients undergoing Laparoscopic Cholecystectomy. It maintains stable hemodynamics during induction and maintenance of general anesthesia compared with propofol. Further studies are needed to validate the findings.

**Trial registration:**

Chictr.org.cn ChiCTR2300071256 (date of registration: 09/05/2023).

## Introduction

For the surgical management of gallbladder disease, laparoscopic cholecystectomy (LC) has been the gold standard due to its less surgical time, minimal trauma, less blood loss, quick recovery, and low infection rate [1]. It is essential to manage hemodynamic fluctuation during surgery. With total intravenous anesthesia (TIVA), where pneumoperitoneum for laparoscopic surgery showed less hemodynamics fluctuation than inhalational anesthesia [2]. Propofol is the most frequently used anesthetics in total intravenous anesthesia due to its quick onset and recovery from anesthesia [3], it also controls perioperative stress and inflammatory reaction. Nonetheless, propofol is associated with a high rate of adverse effects, such as injection pain [4], hypotension, respiratory depression, hypoxemia [5], and propofol infusion syndrome [6]. Therefore, seeking new anesthetic drugs with high efficacy and fewer side effects is essential.

Remimazolam tosilate (RT) is a new ultra-short-acting benzodiazepine with sedative and hypnotic effects [7]. It acts on the gamma-aminobutyric acid (GABA)A receptor. RT is rapidly hydrolyzed in the body to an inactive metabolite and can be antagonized by flumazenil [8]. Previous studies demonstrated that remimazolam can improve hemodynamic stability during induction and maintenance of general anesthesia [9]. The bispectral index (BIS) has been used in previous comparative studies on the effects of remimazolam and propofol on the depth of anesthesia [10–12]. In theory, remimazolam tosilate could be ideal for patients undergoing general anesthesia. However, relevant randomized controlled studies to verify the efficacy and safety of remimazolam during general anesthesia are still lacking.

Therefore, this study aimed to compare the efficacy and hemodynamic stability of remimazolam tosilate during general anesthesia for patients undergoing laparoscopic cholecystectomy, and the occurrence of adverse events of postoperative recovery with those of propofol.

## Methods

### Study design

This was a prospective, single-center, single-blind, randomized controlled trial. The study protocol was approved by the Medical Ethics Committee of the First People’s Hospital of Zunyi, and written informed consent was obtained from all participants. This study was conducted at the First People’s Hospital of Zunyi. It was carried out according to the guidelines of the Declaration of Helsinki and registered with chictr.org.cn (09/05/2023, ChiCTR2300071256; main researcher: Zhuxin Luo). The study design adhered to the 2010 CONSORT statement.

### Participants

A total of 122 patients were scheduled for laparoscopic cholecystectomy under general anesthesia at the First People’s Hospital of Zunyi between May 2023 and November 2023. The inclusion criteria included patients with (1) aged 18 to 60 years, (2) American Society of Anesthesiologists (ASA) physical status I to II, (3) a body mass index (BMI) of 18 to 28 kg/m^2^, and (4) signed the informed consent. Exclusion criteria included (1) respiratory or circulatory dysfunction, (2) severe neuropsychiatric disorders, (3) allergy to or have contraindications with benzodiazepines, opioids, propofol or its ingredients, (4) history of opioid and psychotropic drug dependence, and (5) refusal to sign the informed consent.

Patients were randomly divided into two groups by using a computer to generate a random number list at a 1:1 ratio to the propofol (Group P, *n* = 57) or remimazolam tosilate group (Group RT, *n* = 57). The attending anesthesiologist could not be blinded to group identity due to the difference in the two aesthetics’ color and dosage forms while the patients, operators, and study investigators were blind to group identity. The random group sequence number was placed in sealed envelopes by a nurse who was not involved in the anesthesia. Another anesthesiologist opened the envelope and was aware of the treatment allocation of each patient. Group allocation was revealed only after data collection and analysis.

### Anesthesia and interventions

#### Induction of general anesthesia

No pharmacological premedication was administered before induction, after entering the operating room, standard vital signs monitoring, including the heart rate, non-invasive blood pressure, electrocardiography, pulse oximetry, and the bispectral index (BIS) were performed. In addition, the initial modified observer assessment of alertness/sedation score (MOAA/S: 5 points, responds readily to name spoken in normal tone; 4 points, lethargic response to name spoken in normal tone; 3 points, responds only after the name is called loudly or repeatedly; 2 points, responds only after mild prodding or shaking; 1 point, does not respond to mild prodding or shaking; and 0, does not respond to noxious stimulus) was evaluated [13]. A 20-G intravenous catheter was inserted into a vein on the dorsum of the hand, and lactated Ringer’s solution was administered.

Before the induction of general anesthesia, adequate mask 100% oxygen inhalation at a flow rate of 5 L min^− 1^ was administered for 3 min. In the remimazolam tosilate group (Group RT), remimazolam tosilate (Hengrui, Jiangsu, China; approval number: 221109AU; diluted with 0.9% saline to 1 mg ml^− 1^) was administered as a slow bolus of 0.3 mg kg^− 1^ within 1 min. In the propofol group (Group P), 2.0 mg kg^− 1^ propofol (Enhua, Jiangsu, China; approval number: NBB23J18) was administered as a bolus. The pain was assessed during the drug injection. The time to loss of consciousness (LoC) was recorded. LoC was defined as a MOAA/S score ≤ 1 [14]. While the time to BIS ≤ 60 was recorded. After LoC was attained, sufentanil of 0.5 µg kg^− 1^ and cisatracurium of 0.2 mg kg^− 1^ were administered consecutively. And endotracheal intubation was performed. The baseline mean arterial pressure (MAP), heart rate (HR), and BIS scores of all patients were recorded before induction (T_0_), and these parameters were also recorded at 1 min after intubation (T_1_).

### Maintenance of general anesthesia

During the maintenance of anesthesia, patients were mechanically ventilated with a tidal volume of 6-8mL kg^− 1^. The respiratory rate was adjusted to maintain the end-tidal carbon dioxide concentration at 35–45 mmHg. Remimazolam was adjusted to 1-2 mg kg^− 1^ h^− 1^ and propofol to 4-10 mg kg^− 1^ h^− 1^ based on maintaining the BIS score between 40 and 60. Remifentanil was maintained within the range of 8–15 µg kg^− 1^ h^− 1^ and adjusted as considered appropriate. For hypotension (MAP < 65mmHg or descending 20% basal value), 100 µg of phenylephrine was administered. Atropine (intravenous, 0.5 mg) was administered when bradycardia (HR < 50 beats min^− 1^) occurred. During the surgery, MAP, HR, and BIS were monitored and recorded in both groups at the start of CO_2_ pneumoperitoneum (T_2_), 15 min after the start of CO_2_ pneumoperitoneum (T_3_), 30 min after the start of CO_2_ pneumoperitoneum (T_4_), and the end of surgery (T_5_).

### Recovery from general anesthesia

All drug infusions were terminated at the end of surgery. Simultaneously, the patients were connected to the postoperative patient-controlled intravenous analgesia (PCIA), in which the analgesic formula was 20 µg kg^− 1^ of fentanyl diluted with normal saline to 100 ml. Subsequently, Patients were sent to the postanesthesia care unit (PACU) for extubation (the extubation criteria were as follows: patient awake and following simple commands, 5-second head lift, spontaneous breathing with acceptable oxygenation (regular respiratory rate ≥ 8 breaths min^− 1^, and tidal volume 4–6 mL kg^− 1^).) [15], and neostigmine 1 mg with atropine 0.5 mg would be given if SpO_2_ < 90% lasted for more than 5s and required airway manipulation to recover. After 30 min from the end of remimazolam administration, flumazenil was given if awakening was not yet observed. The time to extubation was recorded. MAP, HR, and BIS were monitored and recorded in both groups at 1 min after extubation (T_6_).

After extubation, the investigator assessed and recorded the following: (1) adverse events, which included intraoperative awareness, nausea and vomiting (PONV), dizziness, delirium, and postoperative low SpO_2_ < 90%; and (2) the time to discharge from PACU.

### Outcomes

The primary outcome was the time to BIS ≤ 60. The secondary outcome included the following: (1) Time to Loc, time to extubation, and time to discharge from PACU; (2) Vital signs of patients especially MAP, HR, and BIS during the surgery; (3) Incidence of adverse events after surgery.

### Sample size calculation

G*Power software (ver. 3.1.9.7, Heinrich-Heine-Universität Düsseldorf, Germany) was used to estimate the sample size required to evaluate the primary outcome. According to our pilot study, the time to BIS ≤ 60 was 87.0 ± 10.1 s in the remimazolam group and 80.2 ± 8.9 s in the propofol group. A sample size of 55 patients in each group was calculated with a type I error of 0.05 and power of 95%, with an effect size of 0.7, and considering a dropout rate of approximately 10%, we finally included 112 patients for analysis in this study.

### Statistical methods

All the analyses were performed with the statistical software packages R 4.3.2 (http://www.R-project.org, The R Foundation, Vienna, Austria) and Free Statistics software (version 1.9.1; Beijing FreeClinical Medical Technology Co., Ltd, Beijing, China). The Shapiro-Wilk test was used to determine the normality of quantitative variables. Normally distributed quantitative variables were expressed as mean ± standard deviation (SD) and were compared using the Student’s *t*-test, repeated measurement variance analysis was used to compare multiple time points within groups. Categorical variables were presented as numbers (percentage) and were compared using a chi-square test, or Fisher’s exact test as appropriate. All tests were two-sided, and *P* < 0.05 was considered statistically significant.

## Results

**Fig. 1:**
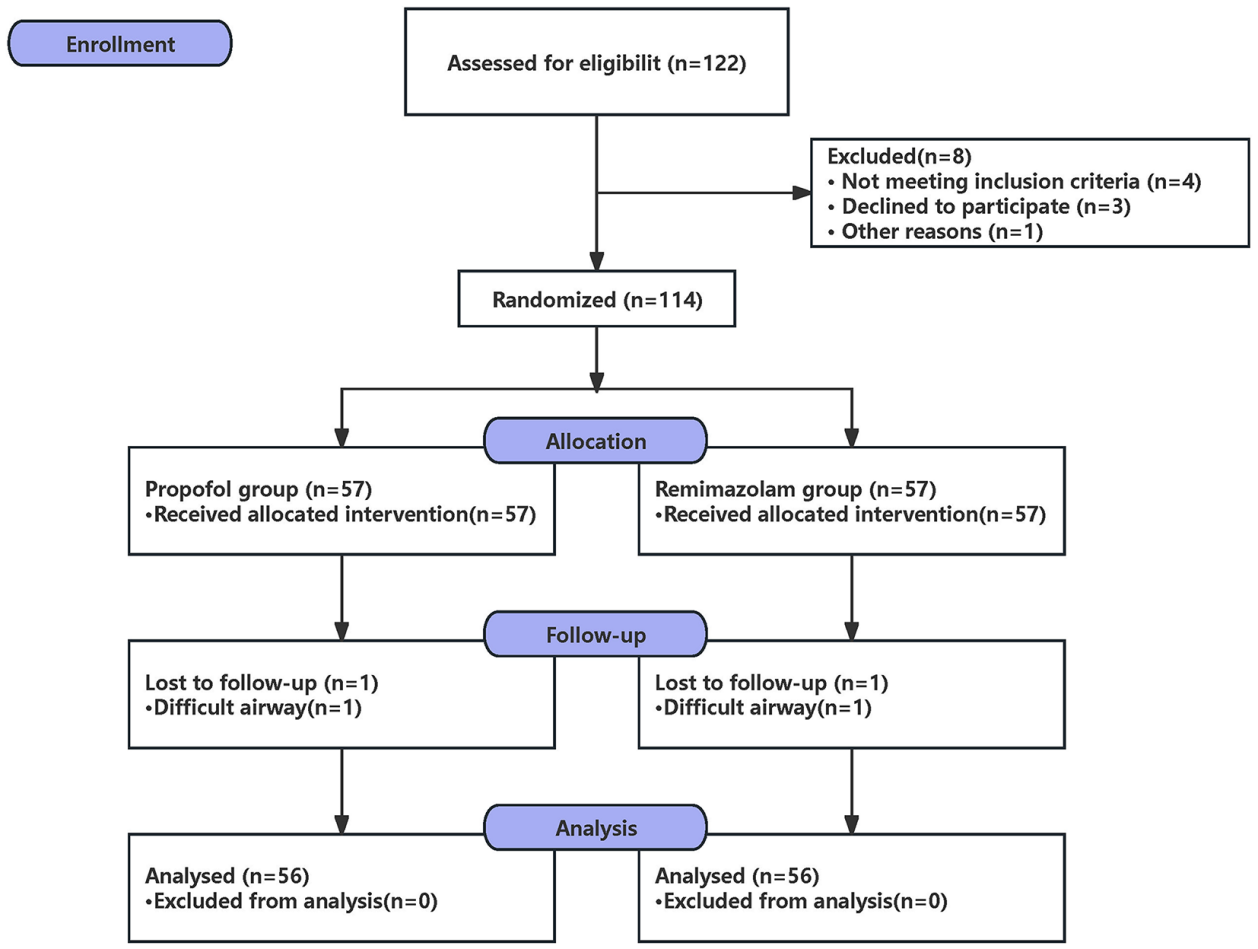
CONSORT flow diagram for patients

From May 2023 to November 2023, a total number of 122 patients were recruited (Fig. 1). Baseline demographics and clinical characteristics were presented in Table 1. There were no significant differences between the two groups in terms of sex, age, height, weight, BMI, history, ASA, the duration of surgery, and the duration of anesthesia (*P* > 0.05).

**Table 1:** Baseline demographics and clinical characteristics

	Group P (*n* = 56)	Group RT (*n* = 56)	*P* value
Sex, n (%)			0.634
Male	10 (17.9)	12 (21.4)	
Female	46 (82.1)	44 (78.6)	
Age (y)	43.0 ± 9.7	42.8 ± 10.4	0.903
Height (cm)	159.3 ± 6.8	158.6 ± 7.5	0.616
Weight (kg)	60.8 ± 8.3	60.2 ± 8.0	0.733
BMI (kg/m^2^)	23.9 ± 2.3	23.9 ± 2.1	0.997
History, n (%)			
Smoking	4 (7.1)	6 (10.7)	0.508
Drinking	3 (5.4)	3 (5.4)	1.000
Allergy	2 (3.6)	1 (1.8)	1.000
CHD	0 (0)	0 (0)	1.000
Hypertension	1 (1.8)	6 (10.7)	0.113
Diabetes	1 (1.8)	2 (3.6)	1.000
ASA, n (%)			0.450
Grade I	48 (85.7)	45 (80.4)	
Grade II	8 (14.3)	11 (19.6)	
Duration of surgery (min)	54.9 ± 17.8	54.2 ± 17.4	0.843
Duration of anesthesia (min)	78.9 ± 19.1	85.0 ± 19.6	0.101

*Note* Data presented as the mean ± standard deviation or numbers(percentage)

*Abbreviations* BMI, body mass index; CHD, Coronary heart disease; ASA, American Society of Anesthesiologists

As shown in Table 2, the time to BIS ≤ 60 in Group RT was slightly longer than that in Group P (Group RT: 89.3 ± 10.7 s; Group P: 85.9 ± 9.7 s, *P* > 0.05). While the time to LoC comparing remimazolam and propofol showed no statistical significance (Group RT: 74.4 ± 10.3 s; Group P: 74.7 ± 9.3 s, *P* > 0.05). The time to extubation in Group RT was significantly longer than that in Group P (Group RT: 16.0 ± 2.6 min; Group P: 8.8 ± 4.3 min, *P* < 0.001). The PACU stay time in Group RT was significantly longer than that in Group P (Group RT: 45.4 ± 10.2 min; Group P: 37.7 ± 10.1 min, *P* < 0.001).

**Table 2:** Induction and recovery times between the two groups

	Group P (*n* = 56)	Group RT (*n* = 56)	*P* value
Start of study drug to LoC (s)	74.7 ± 9.3	74.4 ± 10.3	0.871
Start of study drug to BIS ≤ 60 (s)	85.9 ± 9.7	89.3 ± 10.7	0.087
End of study drug to extubation (min)	8.8 ± 4.3	16.0 ± 2.6	< 0.001
End of study drug to discharge from PACU (min)	37.7 ± 10.1	45.4 ± 10.2	< 0.001

*Note* Data presented as the mean ± standard deviation

*Abbreviations* LoC, Loss of consciousness; BIS, bispectral index; PACU, postanesthesia care unit

**Fig. 2:**
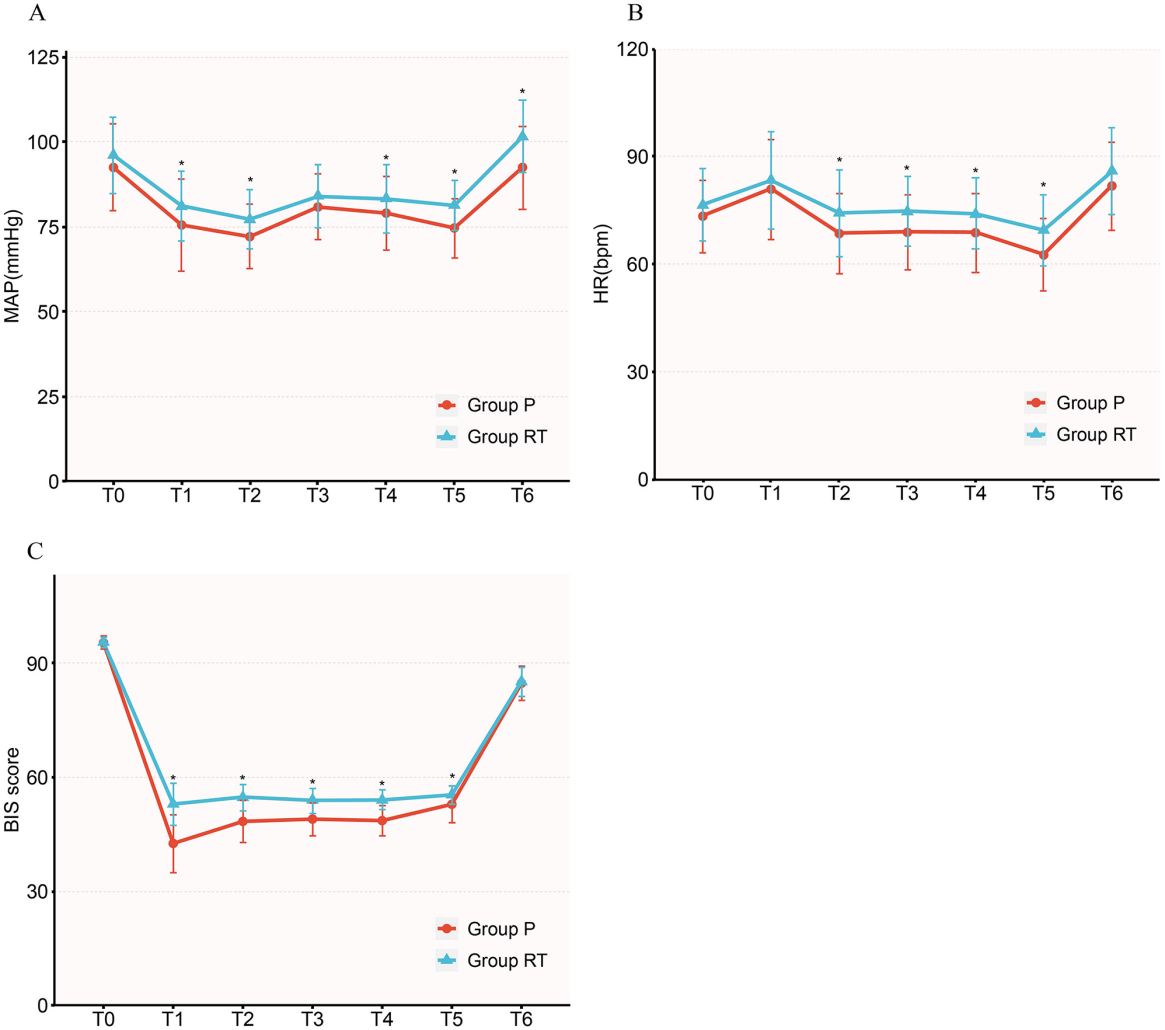
Comparison of hemodynamics during general anesthesia. (**A**) Mean arterial pressure; (**B**) heart rate; (**C**) bispectral index. *Notes* The circles and triangles show the mean, and the error bars show the standard deviation. *Abbreviations* MAP, mean arterial pressure; HR, heart rate; BIS, bispectral index. T_0_, baseline before induction; T_1_, 1 min after intubation; T_2_, the start of CO_2_ pneumoperitoneum; T_3_, 15 min after the start of CO_2_ pneumoperitoneum; T_4_, 30 min after the start of CO_2_ pneumoperitoneum; T_5_, the end of surgery; T_6_, 1 min after extubation. *P < 0.05 was considered to indicate statistical significance between the group comparisons

The MAP, HR, and BIS of 7 time points are summarized in the two groups, as shown in Fig. 2. After anesthesia induction, the MAP at T1-2, and T4-6 tended to be higher in Group RT than that in Group P (*P* < 0.05). Compared with Group RT, the HR at T2-5 in Group P were all significantly reduced (*P* < 0.05). Compared to Group P, Group RT showed less hemodynamics fluctuation in the MAP and HR.

In this study, we also detected BIS during general anesthesia. The BIS scores at T1-5 in Group RT were higher than that in Group P (*P* < 0.05).

We compared the adverse events between the two groups (Table 3). In terms of induction, the incidence of injection pain in Group P was higher than that in Group RT (*P* < 0.05). During anesthesia recovery, the incidence of PONV in Group P was significantly lower than that in Group RT (*P* < 0.05). The incidence of other adverse events such as dizziness and postoperative low SpO_2_ < 90% were similar in both groups (*P* > 0.05). No intraoperative awareness and delirium events occurred in the two groups.

**Table 3:** Adverse events observed

	Group *P* (*n* = 56)	Group RT (*n* = 56)	*P* value
Injection pain, n (%)	42 (75)	0 (0)	< 0.001
Intraoperative awareness, n (%)	0 (0)	0 (0)	1.000
PONV, n (%)	0 (0)	8 (14.3)	0.006
Dizziness, n (%)	10 (17.9)	15 (26.8)	0.257
Delirium, n (%)	0 (0)	0 (0)	1.000
O_2_ saturation < 90%, n (%)	13 (23.2)	7 (12.5)	0.139

*Note* Data presented as the numbers (percentage)

*Abbreviations* PONV, postoperative nausea and vomiting

## Discussion

The purpose of this study was to compare the remimazolam tosilate and propofol used for induction and maintenance of general anesthesia in patients undergoing laparoscopic cholecystectomy, focusing on the efficacy and hemodynamic changes of remimazolam tosilate in general anesthesia, and also investigate the occurrence of adverse events of postoperative recovery. Previous studies have shown that the efficacy and safety of remimazolam are not inferior to those of propofol under general anesthesia [16–18]. In our study, we found that there were no significant differences in the time to LoC and the time to BIS ≤ 60 between the two groups, and no serious events occurred. This result is consistent with the results of previous studies. It verified that remimazolam tosilate might have been as a routine anesthetic for induction of general anesthesia.

Remimazolam tosilate induces sedation by acting on the GABAA receptor, and it is rapidly hydrolyzed and metabolized by tissue esterase enzymes, and it also can be specifically antagonized by flumazenil [19]. Doi et al. [16] demonstrated that without the use of flumazenil, the time to extubation was longer in the remimazolam group than in the propofol group. In contrast, Shi F et al. [20] showed that routine flumazenil injection in the remimazolam group immediately after the end of general anesthesia, the time to extubation and the PACU stay times were shorter than in the propofol. However, some researchers [21, 22] have found that patients may experience re-sedation following the use of flumazenil. In the present study, flumazenil was not routinely administered only when awakening was not yet observed after 30 min from the end of remimazolam tosilate administration. we found that the time to extubation in Group RT (16.0 ± 2.6 min) was significantly longer than that in Group P (8.8 ± 4.3 min), but none of the patients needed to use flumazenil antagonism. Further studies are needed to compare the differences in the recovery time of remimazolam with the administration of flumazenil routinely.

In our study, we confirmed the results of MAP and HR intraoperative changes lower in remimazolam than in propofol. Propofol has obvious inhibitory effects on the hemodynamics. In accordance with the present results, most studies have shown that remimazolam had more stable hemodynamics and a lower incidence of hypotension during surgery [23, 24]. It is important to monitor the depth of general anesthesia, the BIS scores maintained between 40 and 60 being suitable for surgical anesthesia. Myles et al. [25] demonstrated that the use of BIS monitoring can reduce the incidence of awareness during general anesthesia. BIS scores during general anesthesia in remimazolam were significantly higher than those in propofol [20], which is consistent with our results. Although the BIS scores were higher in Group RT, we observed that stable hemodynamics in patients during surgery, without altering the surgical operating conditions for the operator, and there were no instances of intraoperative awareness in patients. RT effectively maintained general anesthesia, providing patients with a satisfactory anesthetic experience while avoiding deep levels of anesthesia. Therefore, our findings indicate that BIS monitoring can be used as an auxiliary technique for assessing the depth of general anesthesia with remimazolam tosilate.

Propofol is widely used for total intravenous anesthesia, but injection pain on propofol is the most important problem in clinical practice [26]. Our finding was consistent with the other studies [17, 27] that no injection pain was found in Group RT. However, the risk of postoperative nausea and vomiting (PONV) was lower when propofol was used [27]. Our results further support the idea of a significantly higher incidence of PONV compared to propofol. The mechanism of PONV during anesthesia requires additional studies to confirm.

Our study had several limitations. First, our study only included participants undergoing elective laparoscopic cholecystectomy at a single center with a limited sample size. Therefore, our results may not be applicable to other procedures elsewhere. Second, we did not compare the differences in the recovery time of remimazolam with the administration of flumazenil routinely, subsequent studies are required to compare the differences in such patients. Third, the follow-up of patients was limited to the postanesthesia care unit, and there was a lack of observation and documentation of long-term complications. Further clinical trials are needed to address the above issues.

In conclusion, remimazolam tosilate can be safely and effectively used for general anesthesia in patients undergoing Laparoscopic Cholecystectomy. It maintains stable hemodynamics during induction and maintenance of general anesthesia compared with propofol. Further studies are needed to validate the findings.

## Data Availability

The datasets generated and/or analysed during the current study are not publicly available due to institutional restrictions but are available from the corresponding author on reasonable request.

## Abbreviations

RT: Remimazolam tosilate
LC: Laparoscopic Cholecystectomy
LoC: Loss of consciousness
TIVA: Total intravenous anesthesia
MOAA/S: Modified observer assessment of alertness/sedation score
BIS: Bispectral index
ASA: American Society of Anesthesiologists
BMI: Body mass index
MAP: Mean arterial pressure
HR: Heart rate
PCIA: Postoperative patient-controlled intravenous analgesia
PACU: Postanesthesia care unit
PONV: Postoperative nausea and vomiting
